# Supra-second interval timing in bipolar disorder: examining the role of disorder sub-type, mood, and medication status

**DOI:** 10.1186/s40345-023-00312-9

**Published:** 2023-10-01

**Authors:** Victória A. Müller Ewald, Nicholas T. Trapp, McCall E. Sarrett, Benjamin D. Pace, Linder Wendt, Jenny G. Richards, Ilisa K. Gala, Jacob N. Miller, Jan R. Wessel, Vincent A. Magnotta, John A. Wemmie, Aaron D. Boes, Krystal L. Parker

**Affiliations:** 1 Department of Psychiatry, The University of Iowa, 200 Hawkins Drive W276GH, Iowa City, IA 52242-1057 USA; 2 Department of Pediatrics, The University of Iowa, Iowa City, IA USA; 3 Department of Psychological and Brain Sciences, The University of Iowa, Iowa City, IA USA; 4 Department of Neurology, The University of Iowa, Iowa City, IA USA; 5 Department of Molecular Physiology and Biophysics, The University of Iowa, Iowa City, IA USA; 6 Department of Neurosurgery, The University of Iowa, Iowa City, IA USA; 7 Department of Radiology, The University of Iowa, Iowa City, IA USA; 8 Iowa Neuroscience Institute, The University of Iowa, Iowa City, IA USA; 9 Institute for Clinical and Translational Science, The University of Iowa, Iowa City, IA USA; 10 Department of Psychology, Gonzaga University, Spokane, WA USA; 11 St. Luke’s Hospital, Cedar Rapids, Iowa, USA; 12 Department of Psychology, St. Mary’s College of Maryland, Maryland, USA

**Keywords:** Cognition, Bipolar disorder, Medication status, Depression, Antipsychotic

## Abstract

**Background:**

Widely reported by bipolar disorder (BD) patients, cognitive symptoms, including deficits in executive function, memory, attention, and timing are under-studied. Work suggests that individuals with BD show impairments in interval timing tasks, including supra-second, sub-second, and implicit motor timing compared to the neuronormative population. However, how time perception differs within individuals with BD based on disorder sub-type (BDI vs II), depressed mood, or antipsychotic medication-use has not been thoroughly investigated. The present work administered a supra-second interval timing task concurrent with electroencephalography (EEG) to patients with BD and a neuronormative comparison group. As this task is known to elicit frontal theta oscillations, signal from the frontal (Fz) lead was analyzed at rest and during the task.

**Results:**

Results suggest that individuals with BD show impairments in supra-second interval timing and reduced frontal theta power during the task compared to neuronormative controls. However, within BD sub-groups, neither time perception nor frontal theta differed in accordance with BD sub-type, depressed mood, or antipsychotic medication use.

**Conclusions:**

This work suggests that BD sub-type, depressed mood status or antipsychotic medication use does not alter timing profile or frontal theta activity. Together with previous work, these findings point to timing impairments in BD patients across a wide range of modalities and durations indicating that an altered ability to assess the passage of time may be a fundamental cognitive abnormality in BD.

**Supplementary Information:**

The online version contains supplementary material available at 10.1186/s40345-023-00312-9.

## Background

Current treatments for bipolar disorder (BD) largely focus on mood symptoms (Schaffer et al. 2015; Scaini et al. 2020; Ashok et al. 2017; Murray et al. 2004). However, changes in cognitive functioning, including deficits in memory, executive function, attention, planning, and timing (Zhou et al. 2018; Frangou 2005; Goldberg 1999; Sole et al. 2012), are common and may even precede a formal BD diagnosis (Hinrichs et al. 2017). Cognitive symptoms are reported by patients with bipolar I disorder (BDI) and bipolar II disorder (BDII), and are present even in a euthymic state (King et al. 2019). As these symptoms are widely reported and linked to lowered quality of life (Brissos and Kapczinski 2008), studies of cognitive symptoms within BD are imperative to a comprehensive understanding of the disorder.

Work has identified timing deficits as one of several cognitive abnormality consistently presented by BD patients (Mahlberg et al. 2008). This also includes abnormalities in attention/working memory, executive function, verbal/visual memory (Andersson et al. 2008). Using finger tapping, auditory temporal bisection, and single-cue delay eye blink conditioning, Bolbecker and colleagues showed sub-second interval timing impairments in BD (Bolbecker et al. 2014a, 2011, 2009a). Using time production and estimation tasks, Bschor and colleagues showed supra-second interval timing deficits in BD ranging from durations of 7 to 109 s. Interval timing depends on diffuse neural networks including the cerebello-thalamo-cortical network and the cortico-striatal network (Grondin 2010; Buhusi and Meck 2005). In BD, abnormalities in nodes of these networks have been previously reported, including frontal cortex, thalamus, and cerebellum (Moorhead et al. 2007; Shaffer et al. 2022; Shinn 2017; Soontornniyomkij et al. 2011; Hibar et al. 2016), providing a mechanistic explanation for the wide-ranging deficits in timing observed in the disorder. Previous work suggests that impairments in interval timing correlate with abnormal fronto-central theta oscillations in patients with SCZ (Singh et al. 2019; Parker et al. 2017). Substantial genetic and symptomatic overlaps between SCZ and BD have been suggested (Murray et al. 2004; Tham et al. 1996). However, it is unclear if the deficits in timing and frontal oscillations observed in SCZ extend to BD.

While extensive work has compared time processing between individuals with BD and other clinical populations or neuronormative controls, there is a paucity of work examining which specific characteristics within BD are linked to timing deficits. Depressive time dilation, the relationship between depressive symptoms and time-perception slowing, is a phenomenon well supported by the literature (Lewis 1932). Additionally, work suggests that manic patients also show alterations in time processing, although works differ in effect directionality (Mahlberg et al. 2008; Bschor et al. 2004). However, is unknown if the specific cognitive deficit of supra-second interval timing observed in BD is linked to mood status at the time of assessment, or if it is a stable characteristic present even in the absence of mood symptoms.

Additionally, while some reports suggest that cognitive impairments in BD can improve in conjunction with mood-symptom treatment (Clark et al. 2002), others suggest that cognitive symptoms may worsen in conjunction with mood treatment (Tham et al. 1996; Clark et al. 2002). Antipsychotic medications may impair measures of general intellectual functioning, working memory, and cognitive set-shifting (Frangou 2005), likely due to reductions in information processing speed. Additionally, anticholinergic burden (ACB), the cumulative effect of taking one or more medications with anticholinergic properties, exerts further negative effects on cognitive functioning (Joshi 2023; Egberts et al. 2021). Although the relationship between medication types, including antidepressants and stimulants, and cognitive functioning in BD has been studied, antipsychotic-use is the only medication related variable with consistently significant impacts on cognition (Frangou 2005). How antipsychotic-use affects the specific cognitive abnormality of interval timing in BD, however, is unknown.

Finally, although it is established that individuals with BD show impairments in timing, work has not explored how this may differ between bipolar disorder sub-types. Given differences in cycling speed and manic episode strength between disorder sub-types (American Psychiatric Association 2013), differences in time perception could be expected. However, work also suggests similar cognitive profiles between BDI and BDII (King et al. 2019), adding a layer of uncertainty to this debate.

To address these questions, we administered a supra-second interval timing task (ITT) to participants with BD and neuronormative controls (CT), while simultaneously recording electroencephalographic (EEG) activity. We hypothesized that individuals with BD would show impaired supra-second ITT performance, in agreement with previous work. As frontal theta activity is related to task-related cognitive processing and given our previous work showing impairments in frontal theta and ITT performance in SCZ patients, we further hypothesized that BD patients would show reduced frontal theta power compared to the CT group during the ITT. Finally, we assessed differences in ITT performance and frontal theta in BD depending on disorder sub-type, depressed mood, or antipsychotic medication use status.

## Methods

### Subjects

The present work stems from a secondary analysis from a clinical trial assessing the safety and efficacy of a novel treatment for bipolar disorder. All measures included in the present dataset were collected during the pre-treatment phase of the clinical trial.

For the BD group, the initial dataset consisted of thirty-one participants. Three subjects discontinued participation in the study before interval timing was assessed. EEG data from four subjects did not meet our outlined data quality criteria (see methods section). This resulted in twenty-four participants (20 females, 4 males) for which data were included in the present work. All BD participants had a DSM-IV diagnosis of BDI (16 subjects) or BDII (8 subjects) and were recruited from the Iowa Longitudinal Database (Tables 1 and 2). Subjects had diagnoses confirmed by a board-certified psychiatrist at the University of Iowa Hospitals and Clinics. Medication status was stable for a minimum of 30 days prior to enrollment and was not altered for the present study (Table 3). Individuals who reported illicit drug use within 6 months of study commencement were excluded from participation.

**Table 1:** Participant demographics

Baseline characteristic	Controls (n = 6)	Bipolar type I (n = 16)	Bipolar type II (n = 8)	p-value
Age				0.948
Mean (SD)	37.2 (10.3)	37.2 (13.5)	35.5 (12.1)	
Sex				0.212
Female	3 (50%)	13 (81.25%)	7 (87.50%)	
Male	3 (50%)	3 (18.75%)	1 (12.50%)	
Race				0.471
White	4 (66.67%)	12 (75%)	6 (75%)	
Black/African American	0 (0%)	3 (18.75%)	0 (0%)	
Asian	1 (16.67%)	0 (0%)	1 (12.50%)	
Other	1 (16.67%)	1 (6.25%)	1 (12.50%)	
Education				0.138
No high school	0 (0%)	2 (12.50%)	0 (0%)	
High school	0 (0%)	5 (31.25%)	3 (37.50%)	
Associate’s/bachelor’s	3 (50%)	7 (43.75%)	5 (62.50%)	
Post-graduate	3 (50%)	2 (12.50%)	0 (0%)	
Handedness				0.126
Right	5 (83.33%)	16 (100%)	8 (100%)	
Left	1 (16.67%)	0 (0%)	0 (0%)	

A one-way ANOVA was used to assess differences in continuous variables, while a Chi-square analysis was used to assess differences between categorical variables

**Table 2:** Comorbidities reported at enrollment for bipolar group

Comorbidity	n (%)
Generalized anxiety disorder	5 (20.8%)
Post-traumatic stress disorder	4 (16.67%)
Panic disorder	2 (8.33%)
Borderline personality disorder	2 (8.33%)
Attention-deficit/hyperactivity disorder	2 (8.33%)
Migraine	1 (4.17%)
Fibromyalgia	1 (4.17%)
Other medical condition	5 (20.8%)

**Table 3:** Medications reported at enrollment for bipolar group

Type of medication	n (%)
Any antidepressant	18 (75%)
SSRI	4 (16.7%)
SNRI	6 (25%)
Atypical antidepressant	11 (45.8%)
Atypical antipsychotic	14 (58.3%)
Lithium	6 (25%)
Benzodiazepine	10 (41.67%)
Stimulant	4 (16.7%)
Anticonvulsant	11 (45.8%)
Opioid	2 (8.3%)

*SSRI* selective serotonin reuptake inhibitor, *SNRI* selective norepinephrine reuptake inhibitor

Mood was assessed via the Montgomery-Asberg depression rating scale (Quilty et al. 2013). A depressed state was defined as a score greater than 10 (Montgomery and Asberg 1979). To analyze the effects of depressed mood and antipsychotic medication-use on the ITT, BD participants were further sub-grouped according to these characteristics. This resulted in a comparison between 8 euthymic patients vs 15 depressed patients. One participant was not included in this analysis as they were in a mixed state. We further compared 14 patients who were on antipsychotic medication vs. 9 patients who were not on antipsychotic medication.

Eight CT subjects were included as a neuronormative comparison group. CT subjects did not have a history of neuropsychiatric disorders. Because of data quality concerns, two CT participants were excluded from the ITT analyses (final n = 6) and one CT participant was excluded from the resting state analyses (final n = 7).

In accordance with federal and institutional guidelines, all procedures including informed consent were approved by the University of Iowa Institutional Review Board and are in accordance with the Declaration of Helsinki.

### Tasks

#### Interval timing task

Concurrent with EEG acquisition, participants performed a supra-second ITT. Participants completed the task sitting in front of a Dell 20” monitor with a 60 Hz refresh rate and 4096 × 2304 screen resolution. White Times New Roman size 40 text appeared on a black background in the middle of the screen. Participants received verbal instructions on how to perform the task from the experimenter and read the same set of instructions on the computer screen. All participants were instructed not to count time in their head. To start each trial, a number indicating the interval to be estimated by the participant (“3” for the short interval/SIT or “12” for the long interval/LIT) appeared on the screen. Participants pressed the space bar to start the trial and to indicate their judgement of the elapsed interval, thus ending the trial. Feedback regarding response accuracy was provided following the button press for every trial. This consisted of the words “You were early/late by” followed by the deviation from the target interval for that trial in sec resolution. The experiment consisted of a total of 80 trials (40 SIT trials & 40 LIT trials) presented in pseudo-random order.

#### Resting state

Resting-state recordings were conducted before the ITT task, lasting 5 min. Participants sat in a chair, were instructed to keep their eyes open, look forward, and let their mind wander.

### Electroencephalography (EEG)

#### EEG acquisition

A BrainVision 64-channel active electrode system with Ag/AgCl electrodes was used to collect EEG (Morrisville, NC). A custom-made electrode cap was utilized, which included electrode placements that are not typical of the International 10–20 system (Klem et al. 1999). Electrodes PO3 and PO4 were substituted by electrodes I1 and I2 which flanked the Iz electrode. The custom-made electrode cap was utilized to collect Iz data for a different experiment which was part of the larger study. Iz data were not analyzed as a part of the present experiment. At the beginning of the recordings, impedances were reduced using high viscosity electrode gel for active electrodes (EASYCAP, Munich, Germany). Impedance for all electrodes was kept at or below 15 kΩ for the duration of the recording. Data were acquired at 500 Hz and referenced online to Pz.

#### EEG analyses

Analyses focused on electrode Fz, as frontal theta oscillations are typically maximal at this site (Shinomia et al. 1994). Frequency bands were defined as: delta (1–4 Hz), theta (4–8 Hz), alpha (8–13 Hz), beta (13–30 Hz), and gamma (30–50 Hz).

Data were preprocessed using custom MATLAB (MathWorks, Natick, MA) scripts based on EEGLAB (Delorme and Makeig 2004) functions. Data were sequentially high-pass filtered at 1 Hz, and low-pass filtered at 50 Hz, the transition bandwidth was set to twice the cutoff frequency (− 6 dB) for cutoff <  = 1 Hz and 25% cutoff frequency for cutoff > 8 Hz. Trials containing nonstereotypic artifacts were removed manually, resulting in exclusion of 18% of trials on average (17% of SIT trials vs 24% of LIT trials). Participants with more than 50% of trials excluded were not included in the final analysis due to data quality concerns. During the ITT, this resulted in the exclusion of two CT participants and four BD participants. During rest, this resulted in the exclusion of one CT participant and four BD participants. Continuous data were re-referenced offline to average voltage. Eyeblinks and saccades were removed using independent component analysis.

Data were epoched as follows: for the interval timing task, data were epoched around the presentation of the timing cue. For the SIT, the epoch ranged from 1 s before cue presentation to 5 s after cue presentation. For the LIT, data were epoched from 1 s before cue presentation to 15 s after cue presentation. For resting-state analyses, continuous data were epoched into 20 s intervals to maintain epochs at approximately the same size between the two tasks. Quantification of band power was conducted using the fast-Fourier transform method. Relative power at each frequency band was defined as the proportion of the overall spectral power distribution occupied by each frequency band, quantified using the MATLAB function trapz.

### Statistical analyses

#### Participant characteristics

Demographic characteristics were compared between individuals in the BDI, BDII, and CT groups using a Pearson’s Chi Square for categorical variables and a one-way ANOVA for continuous variables. Categorical variables were: sex, race, education and handedness. Age was the only continuous variable. Additionally, propensity scores were generated to assess whether age, sex, race, and years of education were associated with the probability of a participant being in the BD group vs. the CT group.

#### Interval timing task performance and band power

To assess performance on the ITT, participants’ time estimates for the SIT/LIT intervals were fit with Gaussian distributions using custom-written MATLAB routines. Timing accuracy and precision were estimated by calculating peak time and coefficient of variation (CV) measures, respectively. The peak time index represents the accuracy of participants’ responses and was calculated using the best fit estimate of the Gaussian distribution. The CV index represents the precision of participants’ responses and was calculated by dividing the response standard deviation by peak time. T-tests were conducted in GraphPad Prism (San Diego, California) to statistically assess performance differences between groups. To examine the relationship between anticholinergic burden to cognition and performance on the ITT, ACB scores were calculated for each patient based on their prescribed medications, as described here (Bishara et al. 2020). A simple linear regression was then conducted comparing each timing index (SIT peak time, SIT CV, LIT peak time, LIT CV) to ACB scores.

Statistical comparisons of power at each oscillation band were compared between groups in GraphPad Prism using t-tests. Multiple comparisons were corrected for using Tukey’s multiple comparisons test.

Statistical outliers were defined as individuals with scores 2 standard deviations above/below their group mean and excluded from the analysis. Mean, standard error of the mean (SEM), and number of outliers excluded for each group are expressed as [GROUP NAME mean ± SEM (number of outliers excluded)].

## Results

### Participant characteristics

**Fig. 1:**
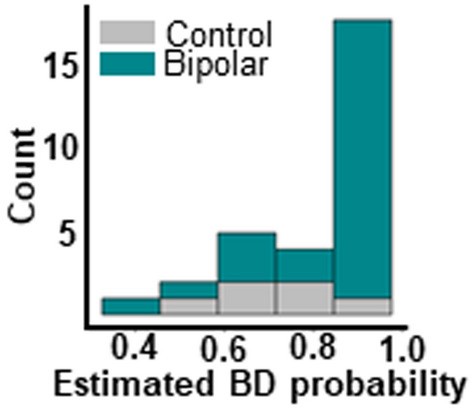
Propensity histogram for neuronormative control and bipolar patient groups. Distribution of control (grey) and bipolar (teal) propensity scores along X axis indicates similar distribution of demographic variables for both groups. Nearly all participants have an estimated probability of being in the BD group that is greater than 0.5 because the majority of our sample belongs to the BD group. This graph indicates that while our groups may not be perfectly balanced, the degree of imbalance between them is not indicative of significant sampling bias

The demographic characteristics age, sex, race education and handedness did not significantly differ between individuals in the BDI, BDII, and CT groups (Table 1). Additionally, although the study population was heavily skewed towards the BD group, propensity scores did not provide strong evidence that participants in the BD vs. the CT groups substantially differed with regards to their age, race, sex, and years of education (Fig. 1).

### Comparison between BD and CT groups

**Fig. 2:**
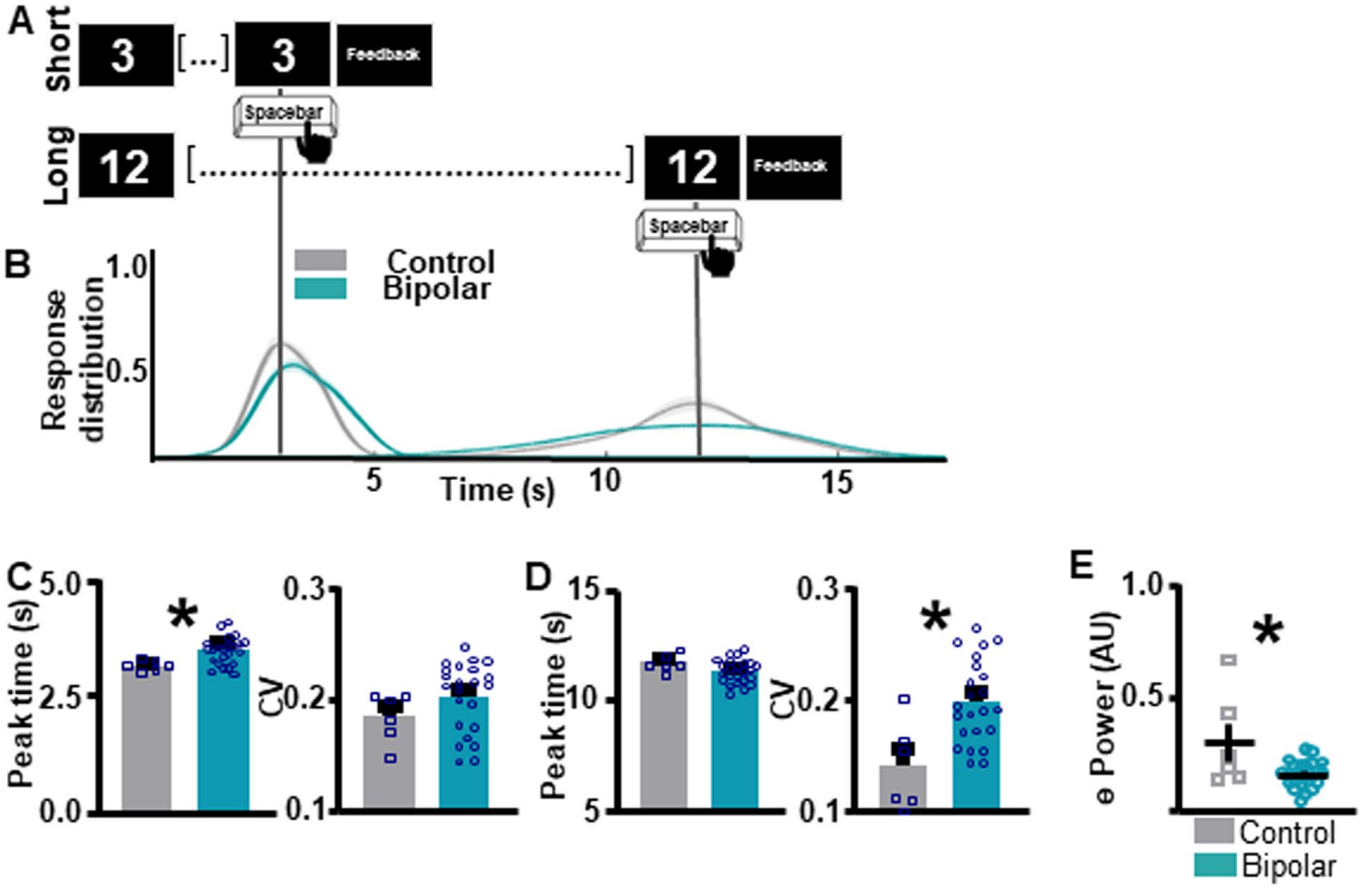
Individuals with bipolar disorder show impairments in supra-second interval timing and abnormal frontal theta compared to neuronormative controls. **A** Schematic diagram of supra-second interval timing task. Trials begin when participants are shown a 3 s or a 12 s timing cue. Participants press the spacebar to indicate their estimation of the target interval. **B** Response distribution for neuronormative controls vs. individuals with bipolar disorder. **C** Individuals with bipolar disorder over-estimate the short interval compared to controls [left]. No differences in response distribution were detected [right]. **D** Individuals with bipolar disorder do not differ from controls in estimation of the long interval duration [left], however, individuals with bipolar disorder have a significantly wider response distribution compared to controls [right]. **E** Individuals with bipolar disorder show lower theta power compared to individuals in the neuronormative control group during the supra-second interval timing task. Mean and standard error of the mean plotted in bar graphs. Dots represent values from individual subjects. * p < 0.05

Individuals with BD show impaired supra-second ITT performance compared to the CT group (Fig. 2A, B). For SIT, individuals with BD show an over-estimation of the target duration compared to the CT group, as quantified by the peak time index (*t*_(27)_ = 2.61, *p* = 0.0146 [BD 3.45 ± 0.0616 (1); CT 3.17 ± 0.0.0466 (0)]; Fig. 2C [left]). Response distribution, quantified by the CV index, did not differ between groups (*t*_(27)_ = 1.33, *p* = 0.192 [BD 0.2032 ± 0.00655 (1); CT 0.184 ± 0.00883 (0)]; Fig. 2C [right]). For the LIT, peak response times did not differ between BD and CT groups (*t*_(28)_ = 1.576, *p* = 0.1262 [BD 11.36 ± 0.1084 (0); CT 11.73 ± 0.1620 (0)]; Fig. 2D[left]). However, individuals with BD showed significantly higher CV indices, indicating higher variability in response times compared to the CT group (*t*_(27)_ = 3.345, *p* = 0.0024 [BD 0.198 ± 0.00778 (1); CT 0.1406 ± 0.161 (0)]; Fig. 2D[right]).

**Fig. 3:**
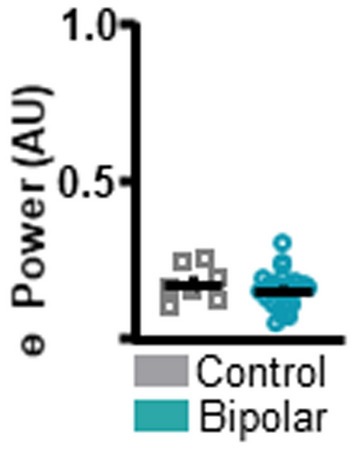
Individuals with bipolar disorder and neuronormative controls do not differ in frontal theta power at rest. To assess resting-state differences in theta power between bipolar disorder and neuronormative control groups resting-state data were analyzed. No differences in resting-state theta power were identified between neuronormative control and bipolar groups. Mean and standard error of the mean plotted in bar graphs. Dots represent values from individual subjects. *p < 0.05

During the ITT, individuals with BD showed lower frontal theta power compared to the CT group (*t*_(27)_ = 2.992, *p* = 0.0059 [BD 0.159 ± 0.0116 (1); CT 0.3000 ± 0.0842 (0)]; Fig. 2E). No differences in power were detected between the BD and the CT groups during the ITT for any other frequency bands (Additional file 1: Fig. S1). For separate examination of theta power during the SIT vs. LIT trials, please refer to Additional file 1: Fig. S2. Although theta power values of a single CT subject are markedly higher than the remainder of the CT subjects, this data point was not excluded, as it does not fit the statistical outlier criteria as described in the methods section. To assess if differences in theta power between BD and CT groups were task-specific, resting-state data were analyzed (Fig. 3). There were no significant differences in theta power between BD and CT groups (*t*_(30)_ = 0.8343, *p* = 0.4107 [BD 0.147 ± 0.009901 (1); CT 0.248 ± 0.0488 (1)]) during rest.

### Comparisons within BD sub-groups

**Fig. 4:**
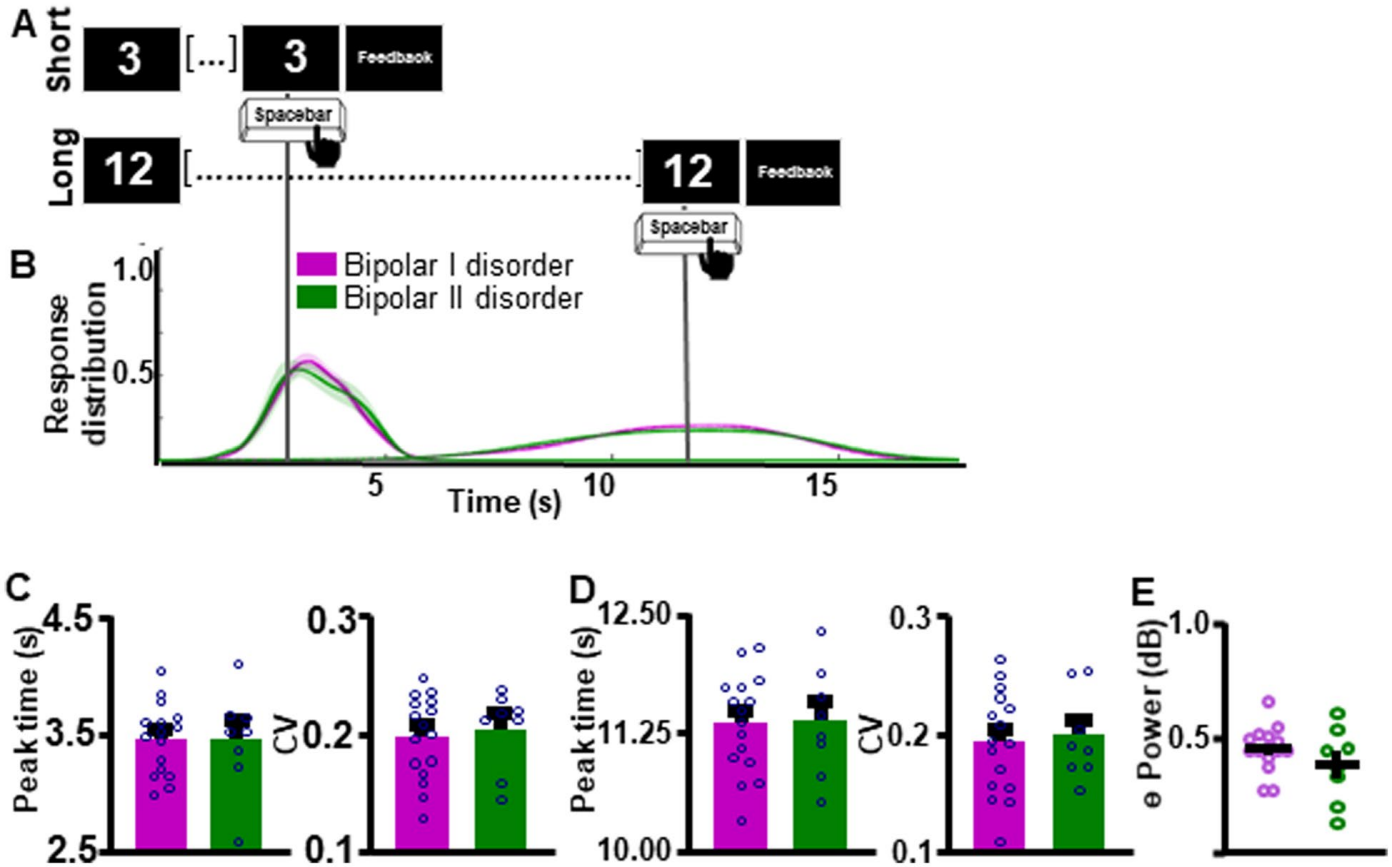
Interval timing performance and frontal theta power do not differ as a function of bipolar disorder sub-type. **A** Schematic diagram of supra-second interval timing task. Trials begin when participants are shown a 3 s or a 12 s timing cue. Participants press a button to indicate their estimation of the target interval. **B** Response distribution for individuals with bipolar I or bipolar II disorder. **C-D** Groups do not differ in time estimation for the short [**C**] or the long [**D**] intervals. **E** Frontal theta power during the ITT did not differ between groups. Mean and standard error of the mean plotted in bar graphs. Dots represent values from individual subjects. *p < 0.05

Within our group of individuals with BD, we first assessed if BD disorder sub-type differentially affected supra-second interval timing ability and associated frontal theta power. Response curves suggest that individuals with BDI and BDII did not differ in their supra-second ITT performance (Fig. 4A, B). Peak time and CV indices did not differ between groups for either the SIT (Peak time: *t*_(22)_ = 0.02449, *p* = 0.9807 [BDI 3.45 ± 0.0760 (0); BDII 3.45 ± 0.153 (0)]; Fig. 4C [left]; CV: *t*_(22)_ = 0.4230, *p* = 0.6764 [BDI 0.198 ± 0.00879 (0); BDII 0.204 ± 0.0120 (0)]; Fig. 4C [right]) or the LIT (Peak time: *t*_(22)_ = 0.9181, *p* = 0.9277 [BDI 11.35 ± 0.130 (0); BDII 11.37 ± 0.206 (0)]; Fig. 4D [left]; CV: *t*_(22)_ = 0.3181, *p* = 0.7534 [BDI 0.192 ± 0.0109 (0); BDII 0.195 ± 0.0130 (0)]; Additional file 1: Fig. S4D [right]) intervals. Additionally, theta power during the ITT did not differ between individuals with BDI vs. BDII (*t*_(21)_ = 1.268, *p* = 0.2188, one BD outlier excluded, Fig. 4E).

**Fig. 5:**
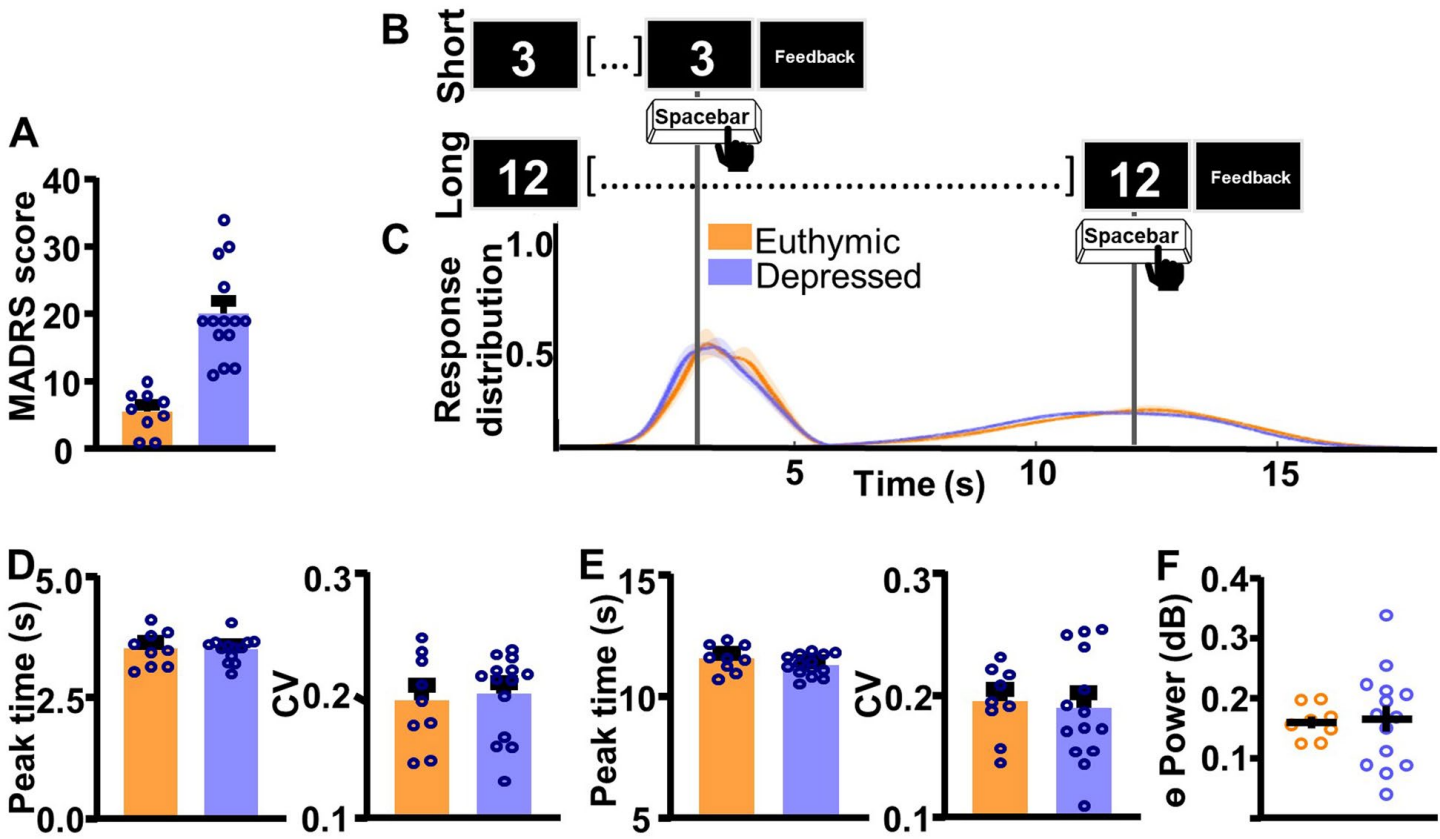
Interval timing performance and frontal theta power do not differ as a function of mood. **A** Average MADRS scores for depressed patients are significantly higher than for euthymic patients. **B** To assess task-wide differences in oscillatory activity data from the whole interval-timing task were analyzed. **C.** Response distribution for individuals with bipolar disorder who were either euthymic or depressed at the time of data collection. **D–E** Groups do not differ in time estimation for the short [**D**] or the long [**E**] intervals. **F** Frontal theta power during the ITT did not differ between groups. *p < 0.05

Next, we assessed if ITT performance and associated frontal theta differed by mood status (i.e. depressed vs. euthymic) within the BD group. Individuals with a MADRS scores above 10 were included in the depressed sub-group (average score 20.07, SEM 1.85). Individuals with a MADRS score below 10 were included in the euthymic sub-group (average score 5.56, SEM 1.04). A t-test suggests a statistically significant separation in MARDS scores between euthymic and depressed individuals (*t*_(21)_ = 5.87, *p* < 0.0001; Fig. 5A). Response curves suggest that supra-second ITT performance does not differ between depressed vs. euthymic individuals (Fig. 5B, C). Peak time and CV indices did not differ between groups for the SIT (Peak time: *t*_(20)_ = 0.05827, *p* = 0.9541 [Euthymic 3.502 ± 0.0725 (0); Depressed 3.510 ± 0.121 (1)]; CV: *t*_(21)_ = 0.3629, *p* = 0.7203 [Euthymic 0.2011 ± 0.00900 (1); Depressed 0.195 ± 0.0124 (1)]; Fig. 5D) or the LIT (Peak time: *t*_(21)_ = 1.333, *p* = 0.1969 [Euthymic 11.30 ± 0.116 (0); Depressed 11.57 ± 0.186 (1)]; CV: *t*_(21)_ = 0.3012, *p* = 0.7662 [Euthymic 0.189 ± 0.0120 (0); Depressed 0.194 ± 0.00967 (1)]; Fig. 5E) intervals. Frontal theta power also did not differ between groups as shown in Fig. 5F (*t*_(20)_ = 0.1963, *p* = 0.8463 Euthymic 0.165 ± 0.0216 (1); Depressed 0.160 ± 0.00999 (1)]).

**Fig. 6:**
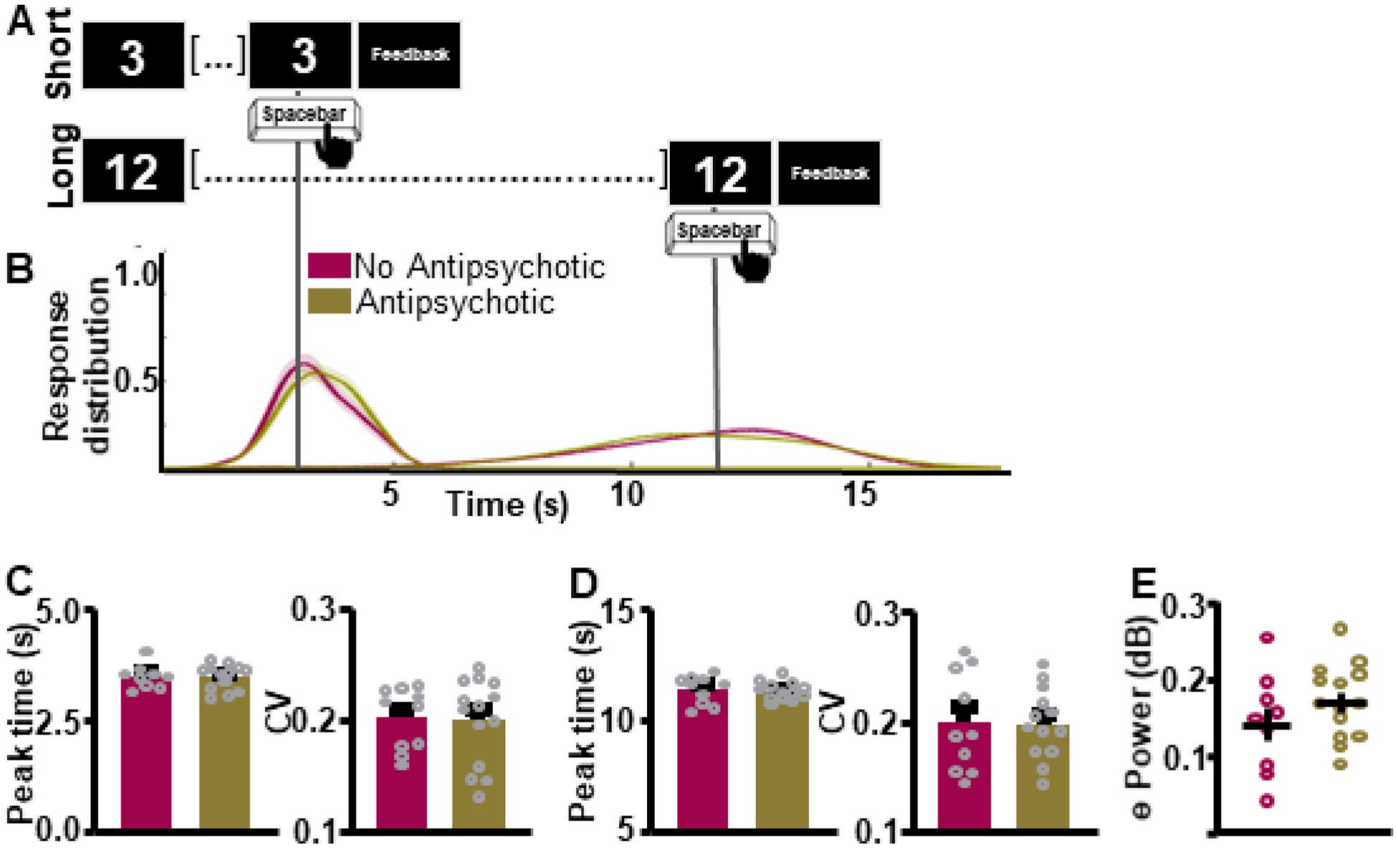
Interval timing performance and frontal theta power do not differ as a function of antipsychotic medication status. **A** Response distribution for individuals with bipolar disorder divided by anti-psychotic medication status. **B–D** Groups do not differ in time estimation for the short [**C**] or the long [**D**] intervals. **E** Frontal theta power during the ITT did not differ between groups. Mean and standard error of the mean plotted in bar graphs. Dots represent values from individual subjects. *p < 0.05

Finally, we assessed if ITT performance and associated frontal theta differed by antipsychotic medication-use within the BD group. Response curves suggest that supra-second ITT performance was not significantly associated with differences in antipsychotic medication status (Fig. 6A, B). Peak time and CV indices did not differ between groups for the SIT (Peak time: *t*_(20)_ = 0.1367, *p* = 0.8927 [BD 3.46 ± 0.0780 (0); CT 3.47 ± 0.0900 (0)]; CV: *t*_(22)_ = 0.1525, *p* = 0.8802 [BD 0.199 ± 0.0103 (0); CT 0.201 ± 0.00898 (0)]; Fig. 6C) or the LIT (Peak time: *t*_(21)_ = 0.2647, *p* = 0.7938 [BD 11.29 ± 0.118 (1); CT 11.35 ± 0.198 (0)]; CV: *t*_(21)_ = 0.02555, *p* = 0.9799 [BD 0.198 ± 0.00886 (1); CT 0.1987 ± 0.01428 (0)], Fig. 6D) intervals. Frontal theta power during the ITT also did not differ between groups Fig. 6E (*t*_(21)_ = 1.284, *p* = 0.2133 [BD 0.171 ± 0.0130 (1); CT 0.140 ± 0.0220 (0)]).

**Fig. 7:**
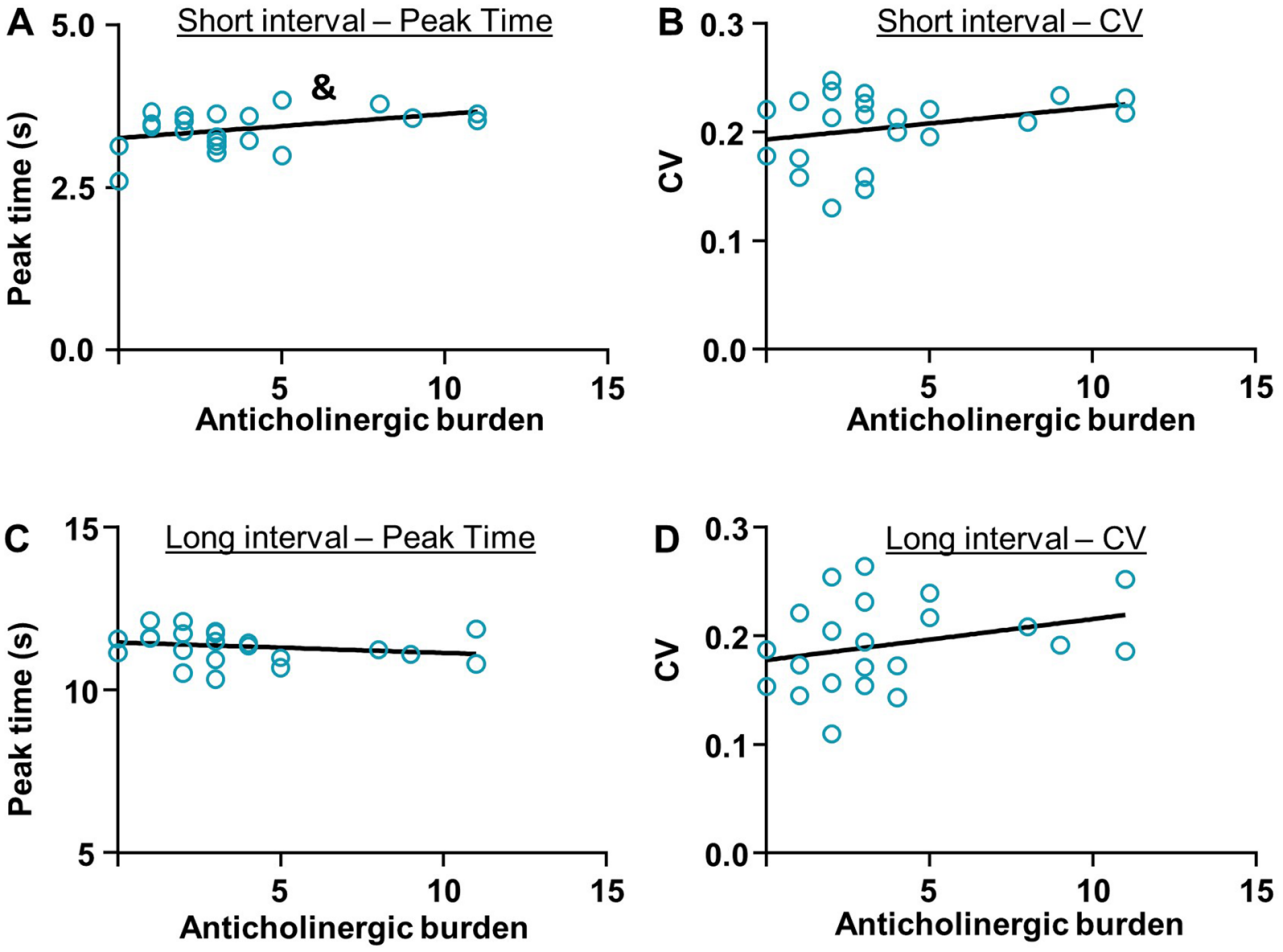
No statistically significant association between anticholinergic burden (ACB) score and performance on the ITT. **A** Marginal association between ACB and the short interval timing index peak time, indicating that the higher the ACB, the more the bipolar participant overestimated the short interval. **B–D** No statistically significant association between ACB and short interval CV (**B**), long interval peak time (**C**), or long interval CV (**D**). & p < 0.10

We also examined the relationship between anticholinergic burden scores and performance on the ITT (Fig. 7). Regression results suggest a marginal association between ACB scores and performance on the ITT for the SIT peak time index (R^2^ = 0.1537, F_(1,20)_ = 3.634, p = 0.0711, Fig. 7A). No association was identified between ACB scores and SIT CV (R^2^ = 0.08906, F_(1,20)_ = 1.955, p = 0.1773, Fig. 7B), LIT peak time (R^2^ = 0.4216, F_(1,20)_ = 0.8804, p = 0.3593, Fig. 7C), or LIT CV R^2^ = 0.08910, F_(1,20)_ = 1.956, p = 0.1772, Fig. 7D).

## Discussion

The objective of the present work was to assess supra-second ITT performance in individuals with BD. Specifically, we were interested in whether BD disorder sub-type, depressive mood, or antipsychotic medication-use altered supra-second interval timing in our cohort of patients. Our results suggest that, although ITT performance and frontal theta were abnormal in the BD group compared to the CT group, within BD sub-groups there were no differences in performance or theta power. Together with previous work indicating that individuals with BD show impairments in supra-second (Mahlberg et al. 2008; Bschor et al. 2004), sub-second (Bolbecker et al. 2014a, 2009a), and implicit motor timing (Bolbecker et al. 2011), our work suggest that an altered ability to assess the passage of time is a fundamental cognitive abnormality in BD.

Cognitive abnormalities are pervasive in psychiatric disorders, although less studied compared to mood dysregulation. For BD alone, the abnormalities in timing described in the present work, are part of a larger set of cognitive difficulties also including irregularities in attention/working memory, executive function, verbal/visual memory (Andersson et al. 2008). Additionally, deficits in interval timing have been previously reported for other neuropsychiatric conditions including SCZ and Parkinson’s disease (Singh et al. 2019; Parker et al. 2017, 2013), indicating the importance of studying the cognitive abnormalities associated with such disorders.

### Trait vs. state abnormalities in BD

Although cognitive impairments are widely reported by BD patients (King et al. 2019), few reports have attempted to triangulate which cognitive impairments may be state vs. trait characteristics of BD. Because clinical characteristics vary between BDI and BDII, and because previous work suggests a link between depressed mood/antipsychotic medication-use and altered temporal processing, we assessed if these characteristics varied within our group of BD patients.

First, our results indicate that supra-second timing performance is not altered as a function of BD disorder sub-type. The extent to which cognitive profiles differ between BDI and BDII is debated in the literature. While some work suggests that BDI presents with more significant cognitive impairments (Sole et al. 2012), other studies suggest similar cognitive profiles between the two sub-groups (King et al. 2019). Indeed, recent work suggests that BDII patients show impairments in attention/working memory, executive function, verbal and visual memory, and motor speed compared to neuronormative controls, just as BDI patients do (Andersson et al. 2008). Our results add to this body of literature, indicating that cognitive impairments in the supra-second interval timing domain, do not differ by BD disorder sub-type.

Additionally, our work suggests supra-second interval timing abilities do not differ between depressed and euthymic BD patients. The lack of distinction between these two groups is interesting given the well-established link between depression and a slowing of time perception in the supra-second domain (Kent et al. 2019). However, previous work suggests that 40–60% of euthymic BD patients may present with neurocognitive impairment (Sole et al. 2012). Indeed, work by Martino and colleagues (Martino et al. 2008) assessed six cognitive domains (attention, verbal memory, language, psychomotor speed, executive function, and facial emotional recognition) in BD and found that 62% of euthymic BD patients showed cognitive impairments, with 40% of patients showing 1 or 2 impaired domains, and 22% of patients showing impairments in 3 to 5 domains. Our findings thus suggest an additional domain—supra-second interval timing—where BD patients show impairments even in the euthymic state, adding to the growing literature indicating cognitive markers as a fundamental characteristic of the disorder.

Finally, our work suggests that antipsychotic medication-use does not alter supra-second timing in BD patients. Past work suggests a negative association between antipsychotic medication-use and IQ in BD (Abrams et al. 1981). Specifically, tests of working memory, set-shifting, and response initiation/inhibition are negatively affected by antipsychotic medication-use (Frangou 2005). However, not all cognitive measures in BD are affected by antipsychotic medication-use, including measures of response planning and general working memory. In the context of this literature, our negative findings concerning antipsychotic use and interval timing are surprising. However, the dependence of timing abilities on working memory and response planning, two cognitive features not altered by antipsychotic use, could explain these findings. Another possibility is that participants were on low antipsychotic doses. Because antipsychotic dose is related to the degree of cognitive impairment, this could explain the lack of group difference. However, dose information was not collected, thus this analysis could not be conducted leaving space for future work.

### Frontal theta during the ITT

The present work identified abnormalities in frontal (Fz) theta oscillations during the ITT in BD patients compared to CT participants. Previous work suggests that ITT performance increases frontal theta power compared to rest. This pattern of activity was indeed detected for the CT group, where visual inspection of ITT vs. resting state graphs suggests that frontal theta power was higher during the ITT (Figs. 2E vs. 3A). However, this pattern was not detected for the BD group, where average power stayed approximately the same during task and rest. Frontal theta oscillatory activity is widely related to cognitive processing for a variety of tasks (Kahana et al. 2001; Amarante et al. 2017). Indeed, compelling theories even propose frontal theta oscillations as a mechanism by which cognitive control may be biophysically realized (Cavanagh and Frank 2014). In this light, our results can be interpreted to suggest a failure in the mechanisms subserving general cognitive functioning and attentional direction in the BD group, which then results in timing deficits.

Previous work suggests that, compared to neuronormative controls, patients with SCZ show abnormal frontal low frequency (delta + theta) activity during the ITT (Singh et al. 2019). Our work suggests that the relationship between abnormal frontal theta and impaired ITT performance may not be a characteristic of SCZ specifically, extending to BD as well. Finally, in SCZ patients, work suggest that abnormalities in theta power in the 500 ms window following timing-cue presentation is related to abnormal supra-second ITT performance (Singh et al. 2019). Although the primary objective of the present work was to analyze ITT performance and theta power within BD sub-groups, not between BD and CT groups, because of this previous SCZ work, secondary analyses were added to identify specific epochs of altered theta power during the task. These analyses were time-locked to cue presentation and response. Results suggest that oscillatory abnormalities in BD were not time-locked to the post-cue interval as they were in SCZ (Additional file 1: Figs. S2B-C and S3B-C). One surprising finding from the present dataset is that individuals with BD showed lower theta power surrounding the response for the long interval (Additional file 1: Fig. S3F), but not the short interval (Additional file 1: Fig. S2F). This parallels performance data where BD patients show altered precision estimates for the long interval (Fig. 2D [right]), but not the short interval (Fig. 2C [right]). These results could indicate that frontal theta power is more closely linked with precision than response accuracy. However, further work is necessary to substantiate this claim.

### Pathophysiology of bipolar disorder

Using timing task performance to triangulate single regions which may be abnormal in BD presents a challenge, as the neuroanatomy of time processing is famously diffuse (Buhusi and Meck 2005) involving the coordinated functioning of multiple brain regions and neurotransmitter systems. One mechanism underlying the altered ITT performance observed in the present work may be the abnormal functioning of the dopamine system in individuals with BD. Indeed, the dopamine hypothesis of BD, which proposes intrinsic dysregulation of dopamine receptor transporter homeostasis (Ashok et al. 2017; Wittenborn 1974), is widely used to explain the pathophysiology of this disorder. Additionally, in other disorders where dopaminergic pathway function is altered, such as SCZ, Parkinson’s, or Huntington’s disease, abnormalities in temporal processing have also been reported (Buhusi and Meck 2005). However, the absence of an effect of antipsychotic treatment on ITT performance weighs against the interpretation of timing deficits being caused by dopaminergic system abnormalities, as this medication class primarily targets the dopamine system.

Another possible mechanism subserving the ITT performance and frontal theta deficits identified in the present work is the well-characterized frontal cortical abnormalities observed in individuals with BD including reductions in cortical grey matter (Moorhead et al. 2007; Michel and Koenig 2018). Indeed, compromised frontal cortical activity has been linked to abnormalities in supra-second interval timing (Buhusi and Meck 2005). This suggests a suggests a failure in the frontal mechanisms subserving time perception in BD patients, expressed electrophysiologically as unaltered frontal theta power and behaviorally as impaired supra-second interval timing.

Although the present work suggests task-wide abnormalities in frontal theta power in the BD group compared to the CT group, it is not ideally set-up to answer when exactly these abnormalities occur. It is possible that abnormal frontal oscillations extend throughout the timing interval, or are limited to the cue, response, or feedback-integration intervals. Our analyses suggest abnormalities locked to the response epoch of the long interval, however, these analyses are preliminary and underpowered (Additional file 1: Figs. S3 and S4). In addition to abnormalities in power, it is possible that a weakening of phase coupling associated with the integration of timing cue processing translates to greater variability in response time. Future work should disentangle this issue.

### Limitations

The present sample is skewed towards BD patients, as the CT group comprises 20% (n = 6 or 7) of the total study population while the BD group comprises 80% (n = 24) of the population. Because of this, the precision of estimates where comparisons between CT and BD groups are made are limited. Indeed, our results should be carefully interpreted as a first step towards understanding supra-second ITT performance in BD, and further, larger studies are needed. However, two indicators within the present data-set point to the reliability of these findings (1) the variability within the control group for most analyses is quite small, smaller than the BD group, in all cases. And (2) we are not the first group to report differences in ITT performance between BD and CT groups, indeed our findings are confirmatory of previous work (Mahlberg et al. 2008; Bschor et al. 2004).

Another factor to consider within the present dataset is that polypharmacy was the treatment plan norm within the patient population, which complicates interpretation of how timing results vary by antipsychotic use. To disentangle this, we calculated an ACB score for each patient. Our regression analysis suggests a weak relationship between the SIT peak time index and ACB score only. However, ACB scores within our data were not normally distributed, and clustered between 1 and 5, complicating interpretation.

Finally, we were unable to assess how mania alters supra-second interval timing performance, as none of our BD patients were in a manic state. This question is of particular interest as results are not consistent within the literature: while some studies suggest that manic patients under-estimate supra-second intervals (Mahlberg et al. 2008), other suggests that manic patients over-estimate such intervals (Bschor et al. 2004). However, this remains an open question for future work.

## Conclusions

Although previous work has established timing deficits in BD, it is unclear if these cognitive abnormalities are due to secondary characteristics associated with BD, such as medication and depressed mood, or if they are a fundamental characteristic of the disorder. In this study, we assessed whether BD sub-type (BDI vs. BDII), depressed mood, or antipsychotic medication-use differentially affected BD patients’ ITT performance and associated frontal theta. Results suggest that ITT performance and frontal theta do not differ between BD sub-types, mood, or antipsychotic medication status. Together with previous work assessing interval timing in BD, these results suggest that an altered ability to assess the passage of time may be a fundamental cognitive abnormality in this disorder.

### Supplementary Information

**Additional file 1: Figure S1.** Power in frequency bands other than theta did not differ between bipolar and control groups. A. To assess task-wide differences in oscillatory activity between bipolar disorder and neuronormative control groups data from the whole interval-timing task were analyzed. B-E. No differences in power were detected between bipolar and control groups for the following frequency bands: delta [B], alpha [C], beta [D], and gamma [E]. Mean and standard error of the mean plotted in bar graphs. Dots represent values from individual subjects. **Figure S2.** Theta power differs significantly and marginally between bipolar and control participants for short and long intervals respectively. A. Theta power is significantly lower in the BD group compared to the CT group during short interval trials. B. Theta power does not significantly differ between BD and CT groups for long interval trials. * p < 0.05, & p < 0.10. **Figure S3.** Time-locked short interval oscillatory activity does not differ between bipolar and control groups. A. Data were epoched around the presentation of the short interval timing cue. B. Averaged spectrogram of individuals in the control group [left] and the bipolar group [right]. Exploratory analyses suggest that oscillatory activity does not differ between the two groups during the whole short interval epoch. C. ROI-based analyses indicate that theta power following the timing cue does not differ between bipolar and control groups. D. Data were epoched around the short interval button press. E. Averaged spectrogram of individuals in the control group [left] and the bipolar group [right]. Exploratory analyses suggest that oscillatory activity does not differ between the two groups. F. ROI-based analyses indicate that theta power prior to the response does not differ between bipolar and control groups. **G.** ROI-based analyses indicate that theta power following the response does not differ between bipolar and control groups. Mean and standard error of the mean plotted in bar graphs. Dots represent values from individual subjects. **Figure S4.** Time-locked theta power surrounding the long interval response is lower in the bipolar group compared to the control group. A. Data were epoched around the presentation of the long interval timing cue. B. Averaged spectrogram of individuals in the control group [left] and the bipolar group [right]. Exploratory analyses suggest that oscillatory activity does not differ between the two groups during the whole long interval epoch. C. ROI-based analyses indicate that theta power following the timing cue does not differ between bipolar and control groups. D. Data were epoched around the long interval button press. E. Averaged spectrogram of individuals in the control group [left] and the bipolar group [right]. Exploratory analyses suggest that oscillatory activity does not differ between the two groups. F. ROI-based analyses indicate that theta power prior to the response was lower in the bipolar group compared to the control group. **G.** ROI-based analyses indicate that theta power following the response was lower in the bipolar group compared to the control group. Mean and standard error of the mean plotted in bar graphs. Dots represent values from individual subjects. * p < 0.05

## Data Availability

The datasets analyzed for the current study will be made available on an individual basis upon reasonable request to the corresponding author.
