# Supplementary material for: Supra-second interval timing in bipolar disorder: examining the role of disorder sub-type, mood, and medication status
Source: Int J Bipolar Disord. 2023 Oct 1;11:32. doi: 10.1186/s40345-023-00312-9 (PMC10542629; doi:10.1186/s40345-023-00312-9)
Supplement: Supplementary file 1 — Additional file 1: Figure S1. Power in frequency bands other than theta did not differ between bipolar and control groups. A. To assess task-wide differences in oscillatory activity between bipolar disorder and neuronormative control groups data from the whole interval-timing task were analyzed. B-E. No differences in power were detected between bipolar and control groups for the following frequency bands: delta [B], alpha [C], beta [D], and gamma [E]. Mean and standard error of the mean plotted in bar graphs. Dots represent values from individual subjects. Figure S2. Theta power differs significantly and marginally between bipolar and control participants for short and long intervals respectively. A. Theta power is significantly lower in the BD group compared to the CT group during short interval trials. B. Theta power does not significantly differ between BD and CT groups for long interval trials. * p < 0.05, & p < 0.10. Figure S3. Time-locked short interval oscillatory activity does not differ between bipolar and control groups. A. Data were epoched around the presentation of the short interval timing cue. B. Averaged spectrogram of individuals in the control group [left] and the bipolar group [right]. Exploratory analyses suggest that oscillatory activity does not differ between the two groups during the whole short interval epoch. C. ROI-based analyses indicate that theta power following the timing cue does not differ between bipolar and control groups. D. Data were epoched around the short interval button press. E. Averaged spectrogram of individuals in the control group [left] and the bipolar group [right]. Exploratory analyses suggest that oscillatory activity does not differ between the two groups. F. ROI-based analyses indicate that theta power prior to the response does not differ between bipolar and control groups. G. ROI-based analyses indicate that theta power following the response does not differ between bipolar and control groups. Mean and standard [file 40345_2023_312_MOESM1_ESM.docx]

# **Additional materials for:** Supra-second interval timing in bipolar disorder: examining the role of disorder sub-type, mood, and medication status

Additional methods

*Time-locked EEG analyses*

Time-locked analyses were conducted on ITT task data via Morlet wavelet convolution. Following pre-processing, data were epoched as follows: for short interval cue presentation, data were epoched from 1 second before cue presentation to 5 seconds after cue presentation. For long interval cue presentation, data were epoched from 1 second before cue presentation to 15 seconds after cue presentation. For responses, data were epoched from 1 second before the response to 1 second after the response. The power spectrum for each trial was convolved with complex Morlet wavelets with a width of 7 cycles, one wavelet for each frequency (1-50Hz). Power values were baseline normalized by conversion to the decibel (dB) scale (10*log10(power epoch/power baseline)). The 250ms window from -400 to -150ms prior to the presentation of the timing cue was utilized as the baseline for both cue and response-centered analyses. For exploratory analyses, the frequency-space assessed was between 1-50Hz. The time-window assessed for the short interval was from cue-onset to 3 s post-cue. The time-window assessed for the long interval was from cue-onset to 12 s post-cue. A simple false-discovery rate correction was used for cell-by-cell comparison of spectral activity over time. For targeted analyses the frequency space assessed was from 4-8Hz (theta band) and the time-window analyzed was the 500ms following the timing-cue onset.

Additional results

**Band-power beyond theta oscillations**

No differences in power were detected between the BD and CT groups during the ITT for the following frequency bands: delta (*t*_(28)_ = 1.607, *p* = 0.1193 [BD 0.417 ± 0.0268 (0); CT 0.324 ± 0.0421 (0)]; Figure S1A), alpha (*t*_(27)_ = 0.1129, *p* = 0.9109 [BD 0.129 ± 0.0119 (1); CT 0.132 ± 0.0179 (0)]; one BD outlier excluded, Figure S1B), beta (*t*_(28)_ = 0.7105, *p* = 0.4832 [BD 0.202 ± 0.0301 (0); CT 0.158 ± 0.0291 (0)]; Figure S1C), and gamma (*t*_(27)_ = 0.6337, *p* = 0.5316 [BD 0.0739 ± 0.00653 (1); CT 0.0854 ± 0.0260 (0)]; Figure S1D).

**Theta power in short vs. long intervals**

The present work was designed to assess task-wide differences in EEG activity, thus, primary EEG analyses do not differentiate between SIT and LIT trials. It is, however, an interesting theoretical point to consider if theta power differs between the two supra-second interval durations. Although the involved neuroanatomy differs between sub- and supra-second intervals [1-3], the question of if neuroanatomy, and if resulting oscillatory activity, differs within different supra-second durations is an interesting and under-addressed one. As a preliminary look into this question, we separately compared theta power for SIT and LIT trials in BD vs CT participants (Figure S2). It is important to note that these analyses are limited by a lack of statistical power (only 40 trials per duration length), and thus must be interpreted as hypothesis-generating for future work the methodology of which is specifically designed to answer this question.

For the short interval, the BD group showed significantly lower theta power than the CT group (*t*_(28)_ = 2.686, *p* = 0.0120 [BD 0.684 ± 0.01416 (0); CT 0.3128 ± 0.09619 (0)], Figure S2A). For the long interval, marginal differences were detected between the two participant groups (*t*_(28)_ = 1.933, *p* = 0.0634 [BD 0.4184 ± 0.02680 (0); CT 0.2920 ± 0.07709 (0)], Figure S2B). While these results may suggest interesting future analyses, sample size limitations limit our ability to confidently infer if theta power differences are originating from one interval length vs the other.

**Time-locked analyses**

Because previous work in SCZ patients suggests that theta power immediately following timing-cue onset correlates to ITT performance [4], in this secondary set of analyses we examine time-locked activity. It is important to note that the present work was designed to assess task-wide differences in EEG activity. Thus, the analyses below should be interpreted as hypothesis-generating for future work that is adequately powered to survive the correction for multiple comparisons associated with cell-to-cell analyses.

Data were epoched surrounding the presentation of the short timing cue (Figure S3A). Averaged spectrograms were created for individuals in the CT group (Figure S3B [left]) and the BD group (Figure S3B [right]). Despite visual differences, exploratory analyses suggest that oscillatory activity does not differ between groups. Targeted analyses suggest that post-cue theta power did not significantly differ between groups (*t*_(24)_ = 1.911, *p* = 0.0680 [BD 0.000558 ± 0.198 (1); CT 0.738 ± 0.224 (0)]; Figure S3C).

Data were next epoched surrounding the button press indicating the participants’ estimation of short interval duration (Figure S3D). Averaged spectrograms were created for individuals in the CT group (Figure S3E[left]) and the BD group (Figure S3E[right]). Exploratory analyses suggest that oscillatory activity did not differ between groups. Targeted analyses suggest that neither pre-response nor post-response theta power differed between groups (*t*_(27)_ = 1.556, *p* = 0.1313 [BD -0.2107 ± 0.187 (1); CT 0.644 ± 0.835 (1)]; Figure S3F; *t*_(25)_ = 0.2206, *p* = 0.8272 [BD -0.328 ± 0.1604 (1); CT -0.2503 ± 0.199 (1)]; Figure S3G).

Data were epoched surrounding the presentation of the long timing cue (Figure S4A). Averaged spectrograms were created for individuals in the CT group (Figure S4B [left]) and the BD group (Figure S4B [right]). Exploratory analyses suggest that oscillatory activity did not differ between groups. Targeted analyses suggest that post-cue theta power did not differ between groups (*t*_(24)_ = 1.663, *p* = 0.1094 [BD -0.326 ± 0.136 (1); CT 0.132± 0.217 (0)]; Figure S4C).

Data were next epoched surrounding the button press indicating the participants’ estimation of long interval duration (Figure S4D). Averaged spectrograms were created for individuals in the CT group (Figure S4E[left]) and the BD group (Figure S4E[right]). Exploratory analyses suggest that oscillatory activity did not differ between groups. Targeted analyses suggest that individuals in the BD group showed lower theta power compared to the CT group before (*t*_(27)_ = 3.946, *p* = 0.0005 [BD -0.0904 ± 0.183 (1); CT 1.48 ± 0.3445 (0)]; Figure S4F) and after the response (*t*_(27)_ = 2.852, *p* = 0.0082 [BD -0.318 ± 0.1843 (1); CT 0.963 ± 0.5405 (0)]; Figure S4G).

Additional Figures


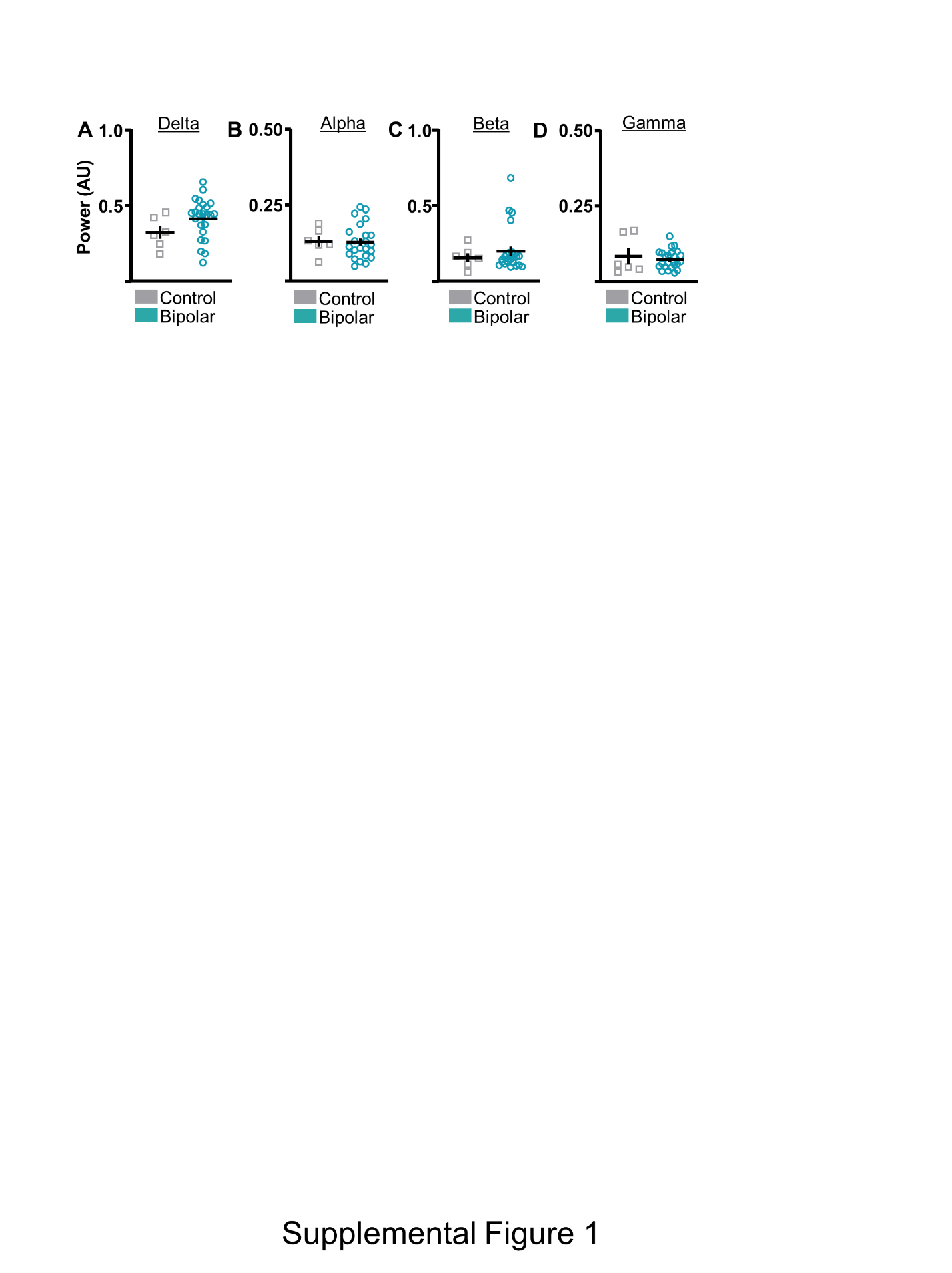


**Figure S1. Power in frequency bands other than theta did not differ between bipolar and control groups. A.** To assess task-wide differences in oscillatory activity between bipolar disorder and neuronormative control groups data from the whole interval-timing task were analyzed. **B-E.** No differences in power were detected between bipolar and control groups for the following frequency bands: delta **[B]**, alpha **[C]**, beta **[D]**, and gamma **[E]**. Mean and standard error of the mean plotted in bar graphs. Dots represent values from individual subjects.


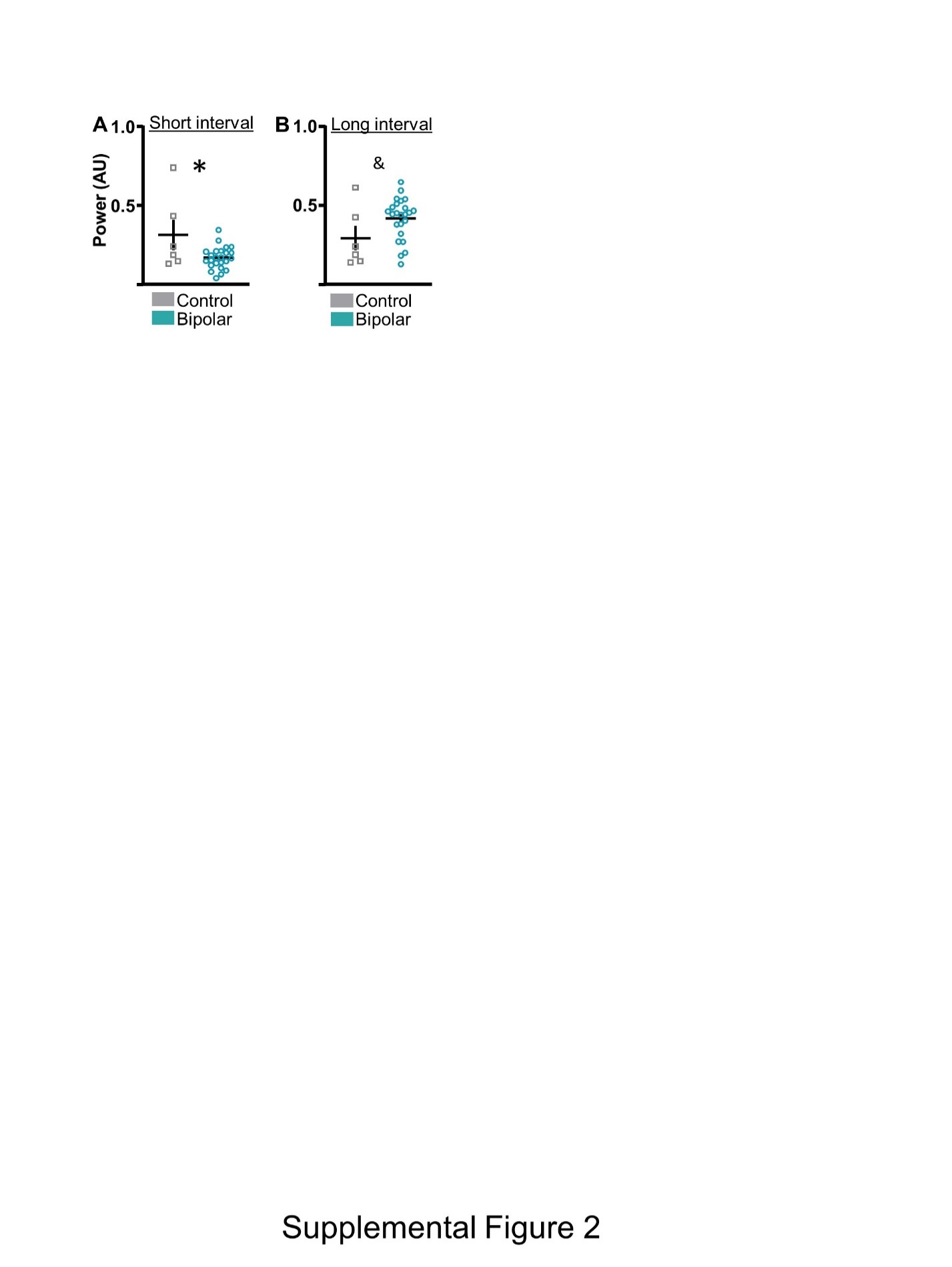


**Figure S2. Theta power differs significantly and marginally between bipolar and control participants for short and long intervals respectively. A.** Theta power is significantly lower in the BD group compared to the CT group during short interval trials. **B.** Theta power does not significantly differ between BD and CT groups for long interval trials. * p < 0.05, & p < 0.10.

**
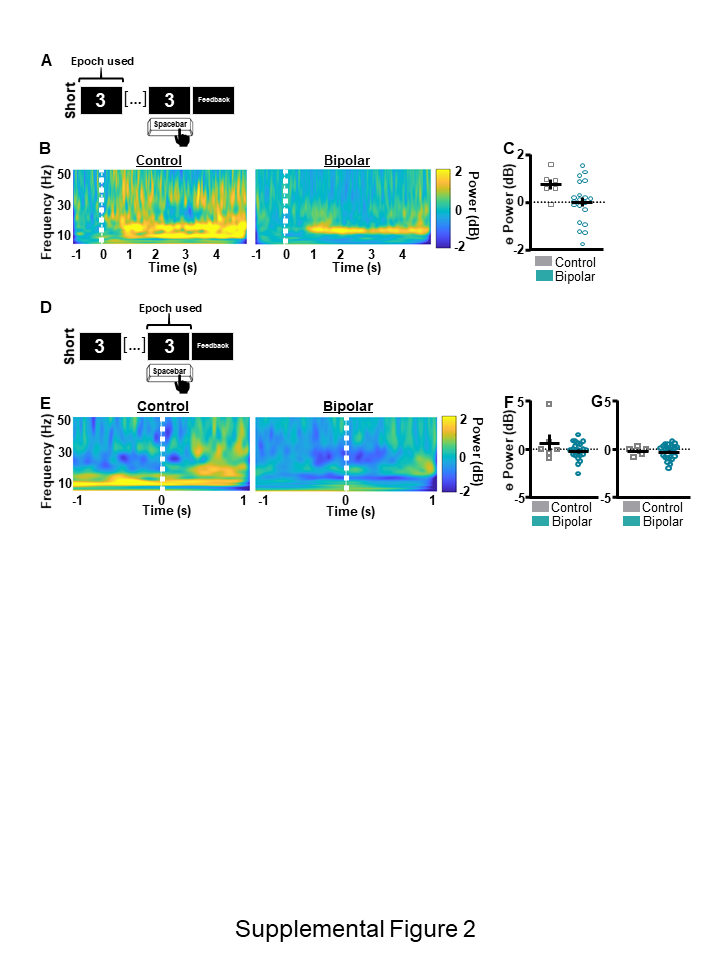
**

**Figure S3. Time-locked short interval oscillatory activity does not differ between bipolar and control groups. A.** Data were epoched around the presentation of the short interval timing cue. **B.** Averaged spectrogram of individuals in the control group **[left]** and the bipolar group **[right]**. Exploratory analyses suggest that oscillatory activity does not differ between the two groups during the whole short interval epoch. **C.** ROI-based analyses indicate that theta power following the timing cue does not differ between bipolar and control groups. **D.** Data were epoched around the short interval button press. **E.** Averaged spectrogram of individuals in the control group **[left]** and the bipolar group **[right]**. Exploratory analyses suggest that oscillatory activity does not differ between the two groups. **F.** ROI-based analyses indicate that theta power prior to the response does not differ between bipolar and control groups. **G.** ROI-based analyses indicate that theta power following the response does not differ between bipolar and control groups. Mean and standard error of the mean plotted in bar graphs. Dots represent values from individual subjects.

**
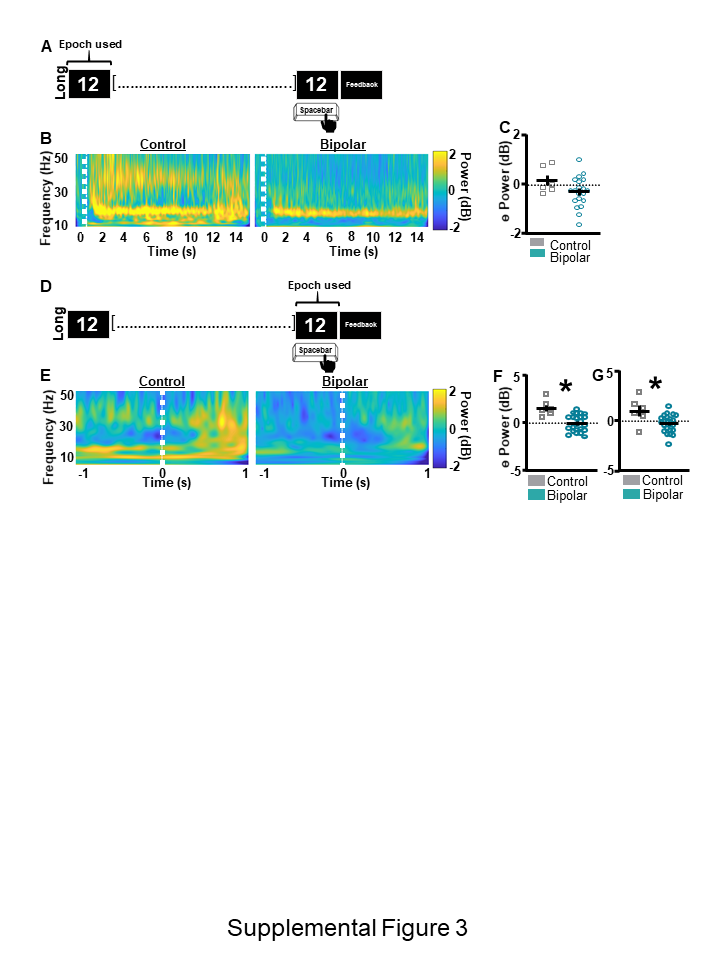
**

**Figure S4. Time-locked theta power surrounding the long interval response is lower in the bipolar group compared to the control group. A.** Data were epoched around the presentation of the long interval timing cue. **B.** Averaged spectrogram of individuals in the control group **[left]** and the bipolar group **[right]**. Exploratory analyses suggest that oscillatory activity does not differ between the two groups during the whole long interval epoch. **C.** ROI-based analyses indicate that theta power following the timing cue does not differ between bipolar and control groups. **D.** Data were epoched around the long interval button press. **E.** Averaged spectrogram of individuals in the control group **[left]** and the bipolar group **[right]**. Exploratory analyses suggest that oscillatory activity does not differ between the two groups. **F.** ROI-based analyses indicate that theta power prior to the response was lower in the bipolar group compared to the control group. **G.** ROI-based analyses indicate that theta power following the response was lower in the bipolar group compared to the control group. Mean and standard error of the mean plotted in bar graphs. Dots represent values from individual subjects. * p < 0.05

Additional References

1. Grondin, S., *Timing and time perception: a review of recent behavioral and neuroscience findings and theoretical directions.* Atten Percept Psychophys, 2010. **72**(3): p. 561-82.

2. Petter, E.A., et al., *Interactive roles of the cerebellum and striatum in sub-second and supra-second timing: Support for an initiation, continuation, adjustment, and termination (ICAT) model of temporal processing.* Neurosci Biobehav Rev, 2016. **71**: p. 739-755.

3. Gallistel, C.R., A. King, and R. McDonald, *Sources of variability and systematic error in mouse timing behavior.* J Exp Psychol Anim Behav Process, 2004. **30**(1): p. 3-16.

4. Singh, A., et al., *Cerebellar Theta Frequency Transcranial Pulsed Stimulation Increases Frontal Theta Oscillations in Patients with Schizophrenia.* Cerebellum, 2019. **18**(3): p. 489-499.
